# General Parenting and Hispanic Mothers’ Feeding Practices and Styles

**DOI:** 10.3390/ijerph18020380

**Published:** 2021-01-06

**Authors:** Thomas G. Power, Jennifer O. Fisher, Teresia M. O’Connor, Nilda Micheli, Maria A. Papaioannou, Sheryl O. Hughes

**Affiliations:** 1 Department of Human Development, Washington State University, Pullman, WA 99164, USA; tompower@wsu.edu; 2 Department of Social and Behavioral Sciences, Temple University, Philadelphia, PA 19140, USA; jofisher1@temple.edu; 3 USDA/ARS Children’s Nutrition Research Center, Department of Pediatrics, Baylor College of Medicine, Houston, TX 77030, USA; teresiao@bcm.edu (T.M.O.); nildam@bcm.edu (N.M.); papaioan@bcm.edu (M.A.P.)

**Keywords:** general parenting styles, feeding styles, feeding practices, Latina, low-income, preschool children

## Abstract

Previous research has shown that general parenting styles, general parenting dimensions, maternal feeding styles, and maternal feeding practices all show specific relationships with the weight status of young children. This study examined the relationships between general parenting and maternal feeding styles/practices in a sample of 187 Hispanic mothers with low incomes. As part of a larger study, mothers of preschool children were recruited through Head Start programs and completed validated questionnaires assessing their general parenting, feeding styles, and feeding practices. Results identified numerous associations between general parenting dimensions and specific feeding practices: i.e., maternal nurturance was positively associated with healthy eating guidance and feeding responsiveness; inconsistency was positively associated with restriction for weight and promotion of overconsumption; follow through on discipline was positively associated with monitoring, healthy eating guidance, and feeding responsiveness; and family organization was positively associated with monitoring and healthy eating guidance. General parenting styles were associated with feeding practices as well, with authoritative mothers showing the highest levels of healthy eating guidance and authoritarian mothers showing the lowest levels of monitoring. There were no significant associations between mothers’ general parenting styles and mothers’ feeding styles. Implications of these findings for the prevention of childhood obesity are considered.

## 1. Introduction

It is well established that general (non-food related) parenting practices and styles are associated with childhood obesity [1,2,3]. In most studies, the authoritative general parenting style (i.e., high demandingness and high responsiveness) is associated with lower child weight status than authoritarian, indulgent, or uninvolved parenting. Moreover, in a meta-analysis of 156 studies, Pinquart [4] found that several parenting dimensions (i.e., individual differences in parent behavior along a single dimension such as nurturance or consistency) were associated with child weight status as well. Children with positive relationships with their parents or whose parents were responsive or showed high levels of maturity demands had lower levels of obesity than those whose parents did not. In contrast, children whose parents were overprotective, used psychological control, or were inconsistent in enforcing rules were at greater obesity risk.

Correlational studies show that parental feeding styles and practices also show significant relationships with child weight status [1,5]. Restriction is usually positively associated with child weight status, whereas pressure to eat often shows a negative relationship [5]. Modeling and food availability sometimes show a negative relationship with child weight status, although the results for these practices are less consistent [1]. Finally, numerous studies show that the indulgent feeding style (high responsiveness and low demandingness) is positively associated with child weight status [6]. Although these results are consistent with the possibility that certain parental feeding styles and practices increase or decrease children’s obesity risk, longitudinal studies show that the direction of effects is often bidirectional, with child weight status predicting parental feeding behavior as well [7,8,9,10].

One possible explanation for the relationship between general parenting and children’s weight status is that general parenting may be related to parental feeding practices and styles in a systematic manner. This possibility has led numerous researchers to examine the relationships between parenting in the feeding and nonfeeding domains. Many of these studies have examined the relationships between Baumrind and Black’s [11] three parenting styles (authoritative, authoritarian, and permissive) and the three feeding practices (restriction, pressure to eat, and monitoring) assessed by the Child Feeding Questionnaire (CFQ) [12]. Consistent findings (i.e., those found in at least two studies) are presented in the Table 1.

As shown in the table, when feeding their children, authoritative parents are more likely to model the consumption of healthy foods and less likely to use the highly controlling strategies of pressuring their child to eat or instrumental feeding (using food as a reward or punishment). Stricter, authoritarian parents, in contrast, are less likely to model consumption and more likely to use the controlling, parent-centered strategies of pressure to eat and restriction. Finally, permissive parents are less likely to monitor their children’s consumption and to model the consumption of healthy foods, and more likely to feed their children to manage their children’s emotions. Surprisingly, they engage in higher levels of restriction than parents showing the other feeding styles—possibly in response to the low levels of self-regulation often found in children of permissive parents [20,21].

Three studies examined the relationships between general parenting dimensions and feeding practices [20,21,22]. McPhie and colleagues [20] found that maternal warmth was positively associated with monitoring children’s food consumption and Vereecker and colleagues [22] found that parental support was positively associated with the use of child-centered feeding practices. Finally, Rodenberg and associates [21] found that parental support was positively associated with encouragement of eating and the overt control of consumption and negatively associated with instrumental and emotional feeding. Psychological control showed the opposite pattern of correlations: negatively correlated with encouragement and overt control during eating and positively correlated with instrumental and emotional feeding.

Two studies examined the relationship between general parenting and parental feeding styles [23,24]. Both examined how general parenting dimensions on the Parenting Dimensions Inventory (PDI) [25,26] differed between parents showing the four feeding styles assessed with the Caregiver’s Feeding Styles Questionnaire (CFSQ) [24]. Table 2 shows results that were consistent across both studies.

Finally, Hennessy and colleagues [23] directly examined the relationship between general parenting styles assessed with the PDI and feeding styles assessed with the CFSQ. Although the Fisher’s exact test showed that there was a statistically significant relationship between general parenting and feeding styles (*p* < 0.05), the results showed very little agreement across these two domains.

Despite the amount of research in this area, the vast majority of studies have involved middle class, European or European American samples. Of the studies identified in the literature review for this paper, only four included significant numbers of ethnic minority parents [15,18,23,24]. Two studies considered feeding styles only [23,24] and the remaining two examined only 3–4 feeding practices [15,18]. Most studies of European or European American parents have examined only a small number of feeding practices as well—the two exceptions being a study of British mothers that supplemented the CFQ with the four subscales of the Parental Feeding Styles Questionnaire (PFSQ) [27] and an online study of predominately European American mothers [14] that examined maternal responses to the 12 subscales of the Comprehensive Feeding Practices Questionnaire (CFPQ) [28].

Given the limited number of studies on the relationship between general parenting and feeding in minority samples, and the focus of most studies on a small number of feeding behaviors, the purpose of the current study was to provide a detailed examination of the relationship between general parenting and parental feeding behavior in a low-income, Hispanic sample—a group at high risk for the development of childhood obesity [29,30]. Although feeding research primarily has focused on European [31,32,33,34], Australian [35,36], and European American [37,38,39] parents and children, research on Hispanic populations is becoming more common [24,40,41,42]. Like studies of European American parents [5], studies of Hispanic parents find a negative association between pressure to eat and children’s body mass index [42,43,44,45]. In contrast, only one [42] of six studies of Hispanic mothers found a significant relationship between restriction and child body mass index z-scores (BMIz) [43,44,45,46,47]. In one of these studies [43], although there was no significant relationship between restriction and child BMIz for Hispanic mothers, this relationship was significant and positive for African American mothers, a result typically found in European American mothers [5]. Similarly, in two studies that used general measures of maternal control during feeding (i.e., measures that combined restriction and pressure to eat) [48,49] there were no significant correlations between maternal control and child BMIz for Hispanics, but the correlations were significant for non-Hispanics.

The findings for restriction parallel studies in the nonfeeding domain where measures of parental restriction and control typically associated with negative child outcomes in non-Hispanic samples show weaker, nonsignificant, or positive relationships in Hispanic samples [50,51,52,53]. Because Hispanic mothers sometimes show high levels of control [51], Halgunseth and colleagues [54] argued that some controlling interactions may not have a negative impact on children because this is one way that Hispanic mothers may show involvement and caring. Consistent with this interpretation is Domenech and colleague’s [55] concept of “protective parenting” in Hispanic families—a style characterized by high demandingness, high responsiveness, and low autonomy granting. Hispanic mothers also show greater control than non-Hispanics in feeding: both restriction [56] and pressure to eat [56,57]. Finally, several studies of feeding style [24,44,58] have shown that although the indulgent feeding style puts Hispanic children at the highest risk for obesity, the authoritarian feeding style is associated with the lowest child obesity rates—suggesting that this highly controlling feeding style may be protective against childhood obesity in this population.

The current study examined a wide range of parenting practices and styles in both the feeding and non-feeding domains. Four general parenting dimensions (i.e., nurturance, inconsistency, follow through on discipline, and organization) along with four general parenting styles (authoritative, authoritarian, indulgent, and uninvolved) were assessed with the PDI; four second order-factors [59] derived from the 12 subscales of the CFPQ examined feeding practices (monitoring, restriction for weight, promotion of overconsumption, and healthy eating guidance); and four feeding styles (authoritative, authoritarian, indulgent, and uninvolved) were assessed with the CFSQ.

Based on the literature reviewed above, it was hypothesized that: (1) nurturance would be positively associated with healthy eating guidance and parental responsiveness in feeding; (2) inconsistency would be positively associated with promotion of overconsumption and demandingness in feeding; (3) follow through on discipline would be positively associated with monitoring, healthy eating guidance, and feeding responsiveness; and (4) family organization would be positively associated with monitoring and healthy eating guidance. Regarding the general parenting styles, it was predicted that: (5) authoritative mothers would show the highest level of monitoring and healthy eating guidance; (6) authoritarian mothers would show the highest level of restriction for weight and promotion of overconsumption; (7) authoritative and indulgent mothers would show the highest levels of feeding responsiveness; and (8) authoritarian mothers would show the highest level of feeding demandingness.

## 2. Materials and Methods

### 2.1. Participants

A total of 187 Hispanic parents and their preschool children, recruited through Head Start centers in Houston, Texas, participated. These data were collected as part of a larger longitudinal study of self-regulation, parent–child interaction, and childhood obesity in a sample of Hispanic parents with low incomes [60]. Only the data from the first time point were used for this paper. The person responsible for feeding the preschool child when he or she was not at school was invited to participate. All except two participants were mothers; two were grandmothers. Participants are therefore referred to as “mothers” in the text that follows. Additional details on the methods and measures can be found in Hughes et al. [60]. The procedures were approved by the Institutional Review Board at Baylor College of Medicine (H-26796). Mothers signed consent forms before participating; children provided verbal assent. Participants received $90 for participating at the first time point of this study: $25 for the first day and $65 for the second day. Descriptive information on the sample is provided in Table 3.

### 2.2. Procedures

At the first time point, mothers and children came to the study laboratory on two separate days about a week apart. On the first day, mothers and children completed tasks not analyzed here. On the second day, children completed two executive functioning tasks, the delay of gratification task prior to consuming a standard meal, and the eating in the absence of hunger task. These tasks are described below. Mothers were instructed to not feed their children prior to coming to the study laboratory. Mothers completed a series of questionnaires, including those analyzed for this paper. Trained research staff measured the heights and weights of the mothers and children as well. Tasks were completed in either English or Spanish depending upon the child’s preference: 77% of the tasks were completed in Spanish. Additional details can be found in Hughes et al. [60].

### 2.3. Measures

Two sets of measures from the larger Hughes et al. [60] study are described below. The primary measures were the PDI-S [25,26] to measure general parenting styles, the CFPQ [28] to assess feeding practices, and the CFSQ [24] to measure feeding styles. Unfortunately, the “amount of control” subscale of the PDI (one of the two PDI subscales typically used to assign mothers to general parenting styles—[23,61,62]) was not reliable in this sample (see below). Therefore, we validated a new method used to assign mothers to general parenting styles in this study. We accomplished this by examining the relationships between general parenting style and several additional measures from the larger Hughes et al. [60] study (i.e., child self-regulation measures). These secondary measures are briefly described below. More details can be found in Hughes et al. [60].

#### 2.3.1. Parenting Dimensions Inventory: Short Form (PDI-S)

General parenting was assessed with the primary subscales of the PDI-S [25,26]. These scales were derived from 17 items on 6-point Likert scales: nurturance (e.g., “I encourage my child to talk about his or her troubles,” 6 items), inconsistency (e.g., “There are times I just don’t have the energy to make my child behave as he or she should,” 4 items), follow through on discipline (e.g., “Once I decide how to deal with a misbehavior of my child, I follow through on it,” 3 items), and organization (e.g., “Our family is organized,” 4 items). Amount of control was assessed with five dichotomous items. For each item, mothers chose one of a pair of opposing statements that best described her approach to parenting—one reflected the level of control expected of a permissive parent (e.g., “Children need more freedom to make up their own minds about things than they seem to get today”) and one reflected the level of control expected of an authoritative or authoritarian parent (e.g., “Children need more guidance from their parents than they seem to get today”). Coefficient alphas for the PDI subscales in the current sample were: nurturance (0.74), inconsistency (0.66), follow through on discipline (0.71), organization (0.64), and amount of control (0.03). Because of the low alpha for amount of control, this scale score was not used in the current analyses.

The PDI has predicted a number of child outcomes in various Spanish-speaking samples including achievement [63], social competence [64], smoking initiation [62], and weight status [65]. In addition to examining the correlates of the parenting dimensions on the PDI-S, we also assigned mothers to the authoritative, authoritarian, indulgent, or uninvolved parenting styles using a modification of the approach used in Hood et al. [61], Olvera and Power [65], and Hennessy et al. [23]. The approach for assigning mothers to general parenting styles is described in the preliminary analyses section below.

#### 2.3.2. Comprehensive Feeding Practices Questionnaire (CFPQ)

The CFQP assessed a range of parental feeding practices with 49 items on 5-point scales [28]. It is a widely used feeding questionnaire. Although it was developed and validated on a middle-class, European American sample, it has been used successfully with Hispanic samples [66,67]. The questionnaire yields scores on 12 separate subscales. However, in an exploratory factor analysis in the current sample [59], five factors were identified: monitoring (e.g., “How much do you keep track of the sweets—candy, ice cream, cake, pies, pastries—that your child eats? 4 items), restriction for weight (e.g., “I restrict the foods my child eats that might make him/her fat,” 7 items), promotion of overconsumption (e.g., “If my child eats only a small helping, I encourage him/her to eat more,” 8 items), healthy eating guidance (e.g., “I discuss with my child the nutrition value of foods,” 12 items), and healthy eating variety (e.g., “I encourage my child to try new foods,” 3 items). Coefficient alphas in the current sample for these five subscales ranged from 0.73 to 0.87 [59].

#### 2.3.3. Caregiver’s Feeding Styles Questionnaire (CFSQ)

The CFSQ is a 19-item questionnaire where respondents indicated the degree to which they use various child-centered practices (e.g., asking questions, providing reasons, and allowing choice, 7 items) and parent-centered practices (e.g., food as a reward, hurrying the child, spoon-feeding, 12 items) when feeding their child [24]. These items were used to derive measures of demandingness (i.e., the degree to which caregivers encourage their child to eat) and responsiveness (i.e., the relative use of child-centered versus parent-centered strategies). Scores on demandingness and responsiveness were used to assign caregivers to one of four feeding styles: authoritative (high demandingness and high responsiveness), authoritarian (high demandingness and low responsiveness), indulgent (low demandingness and high responsiveness) and uninvolved (low demandingness and low responsiveness). This instrument was developed using low-income samples [24] and its reliability and validity have been demonstrated in numerous studies of ethnic minority samples (see Hughes and Power [6] for a review). Coefficient alphas in the current sample were 0.66 for child-centered strategies and 0.84 for parent-centered strategies.

#### 2.3.4. Child Body Mass Index (BMI) z-Scores

Children were weighed and height measurements taken using standard procedures [68]. Measurements were recorded to the nearest 0.1 kg for weight and 0.1 cm for height. Age- and gender-specific BMI *z*-scores were calculated using the Center for Disease Control and Prevention Reference Standards [69].

#### 2.3.5. General Self-Regulation (Measures for Validation of New PDI Scoring Only)

Children’s general self-regulation was assessed by both maternal questionnaires and laboratory tasks with the children. These included maternal ratings of children’s emotion regulation (Emotion Regulation Checklist, ERC [70]) and children’s effortful control (Children’s Behavior Questionnaire, CBQ [71]). The alpha for the effortful control subscale of the CBQ in this sample was 0.69. Because the original ERC scoring did not yield acceptable alphas in this sample (liability 0.56, emotion regulation 0.45), we ran a confirmatory factor analysis based upon an a priori examination of the item content. Details of this analysis are provided in Olivera [72]. A review of the questionnaire showed that although the questionnaire assessed five constructs, only two had alphas of 0.60 or higher in this sample: frustration (0.60) and positive social regulation (0.60). A confirmatory factor analysis with these two correlated factors (*r* = −0.30) showed excellent fit: chi square (7) = 11.43, ns, *CFI* = 0.967, *RMSEA* = 0.059, and *TLI/NNFI* = 0.929. Although the alphas for these subscales were low, Clark and Watson [73] argued that mean interitem correlations are more appropriate measures of internal consistency when the number of items is small. This value was 0.23 for each of these subscales in the current sample, within Clark and Watson’s [73] suggested range of 0.15 and 0.50. Given this recommendation and the fit of the confirmatory factor analysis, we decided to retain the frustration and positive social regulation subscales for the current analyses.

The laboratory tasks assessing general self-regulation were two executive functioning tasks: the tapping task [74] and the Flexible Item Selection Task, FIST [75]. The tapping task assessed behavioral inhibition. In a series of 16 trials, the experimenter hit a wooden block with a dowel once or twice. The child was instructed to hit the block twice when the experimenter hit it once and hit it once when the experimenter hit it twice. The child must therefore inhibit his or her automatic response (i.e., imitation) and replace the behavior with an incompatible one. The FIST is a measure of cognitive flexibility. On 15 trials, children were shown pictures of three different objects that varied along various dimensions (i.e., color, shape, and size). On each trial, children were told to point to two objects that were alike in “one way” and then told to point to two objects that were alike in “another way.” The number of correct answers on the tapping and FIST tasks were positively correlated in the current sample, *r*(182) = 0.43, *p* < 0.001; therefore, these scores were standardized and summed to create a measure of executive functioning.

Children also completed a delay of gratification task [76]. This task assessed children’s ability to resist the temptation of an immediate, smaller reward in order to wait for a larger reward. Children were presented with two plates of snacks, one with a small amount and one with a large amount. They were left alone in a room with the snacks and a bell. Children were told that if they could wait until the experimenter returned, they could have the larger pile of snacks; if they could not wait, they were told to ring the bell and the experimenter would return and they could have the smaller pile. The distribution of waiting times for this 7-min task was bimodal (reflecting a very short wait time versus waiting the entire time); therefore, data were recoded into three wait values: 1 = less than a minute (*n* = 85); 2 = between one and seven minutes (*n* = 28); and 3 = waited the entire time (*n* = 67). Although a small amount of food was used in this task, it was classified as a measure of general self-regulation because it is considered a measure of “hot” executive functioning. In the current sample, it was positively correlated with the composite measure of executive functioning described above, *r*(176) = 0.43, *p* < 0.001, and not associated with maternal report or task-related measures of eating self-regulation [60].

#### 2.3.6. Eating Self-Regulation (Measures for Validation of New PDI Scoring Only)

Children’s eating self-regulation was measured by both maternal questionnaires and child tasks as well. Three subscales of the Children’s Eating Behavior Questionnaire [77] provided maternal ratings of children’s self-regulation in the eating domain. Two subscales assessed poor regulation (emotional overeating and food responsiveness); one subscale assessed good regulation (satiety responsiveness). Coefficient alphas for these three subscales in the current sample were 0.70, 0.80, and 0.68, respectively. The laboratory task was the Eating in the Absence of Hunger (EAH) task [78]. In this task, children were first provided with a complete meal accounting for 40% of the daily food requirements for a four- to five-year-old. The child was then left alone for 10 min with a tray of sweet and savory snacks and age-appropriate toys. The child was told that he or she could play with the toys and eat as much or as little of the snacks as he or she would like. The snacks were weighed before and after the task and the number of kilocalories consumed in the absence of hunger was calculated. The final scores across the children were highly positively skewed; therefore, data were recoded into three values: 1 = less than 20 kilocalories (*n* = 37); 2 = 20 to 125 kilocalories (*n* = 74); 3 = greater than 125 kilocalories (*n* = 75). High values reflected lower levels of children’s eating self-regulation.

### 2.4. Data Analyses

Given the poor alpha for the amount of control subscale of the PDI in this sample, correlations and descriptive statistics were examined for the five items in this scale and an alternative approach to assigning mothers to parenting styles was developed (this approach is described in the preliminary analyses section below). To further validate the PDI in this sample, and to validate the revised approach to assigning mothers to parenting styles, multiple linear regressions were run, one for each of the self-regulation variables described above (i.e., positive social regulation, frustration, effortful control, executive functioning, delay of gratification, emotional overeating, food responsiveness, satiety responsiveness, eating in the absence of hunger, and child BMI *z*-score). The predictor variables in these regressions were the four PDI subscales (excluding amount of control): nurturance, inconsistency, following through on discipline, and organization. The four predictors were entered simultaneously. We did not enter the predictors in a step-wise fashion because we wanted to examine the independent effects of each parenting dimension and had no theoretical reason for entering the predictors in a particular order (i.e., we did not see any of the parenting dimensions as more important than the rest, so they all were treated equally). After assigning mothers to a general parenting style, a series of analyses of variance was run predicting the same child variables that were examined in the regressions. Significant main effects of parenting style were followed up with Bonferroni tests.

The primary analyses for the study examined the relationship between the general parenting variables and the measures of feeding practices and styles. These were multiple linear regressions predicting the five CFPQ subscales (monitoring, restriction for weight, promotion of overconsumption, healthy eating guidance, and healthy eating variety) and the two CFSQ subscales (demandingness and responsiveness). The PDI predictors were the same as in the regressions described above. Finally, chi square analyses were used to examine the relationship between general parenting styles on the PDI and feeding styles on the CFSQ.

## 3. Results

### 3.1. Preliminary Analyses

Descriptive statistics of the study variables are presented in Table 4. Examination of these statistics showed that one variable, the healthy eating variety subscale of the CFPQ, showed very little variance (93% had a value of 4.0 or greater on a 5-point scale), so this variable was dropped from subsequent analyses.

Because the coefficient alpha for the amount of control subscale on the PDI was unacceptable in this sample (0.03), and standard practice for assigning parents to general parenting styles is to conduct median splits on the nurturance and amount of control subscales [23,61,65], an alternative approach to assigning parents to parenting styles that did not use the amount of control scale had to be developed. We did this by first examining the correlations between the five amount of control items on the PDI (and their frequency distributions) to determine if any of the items on this subscale could be used for this purpose. Only one correlation between the items was significant and it was very small, *r*(185) = 0.14, *p* < 0.05. Examination of the frequency distributions of the items explained why some of the items were poor indicators of this construct. For two items, almost all of the mothers chose the more controlling option: “Children need more guidance from their parents than they seem to get today” (99.5%) and “It is important to set and enforce rules for children to grow up to be healthy adults” (96.8%). Surprisingly, the two obedience items showed an inconsistent pattern of responding, suggesting that mothers misunderstood at least one of the items. This is indicated by the tendency of the majority of the mothers to choose the non-obedient choice (56.1%) on one item (“Nowadays, parents place too much emphasis on obedience for their children”) and the obedient choice (88.7%) on the other (“I care more than most parents I know about having my child obey me”). The correlation between these two items was nonsignificant, *r*(184) = 0.03.

This left only one item on the amount of control scale that had sufficient variability and mothers seemed to understand: “I try to prevent my child from making mistakes by setting rules for his/her own good” (77.0%) versus “I try to provide freedom for my child to make mistakes and to learn from them” (23.0%). Because this item clearly reflects an expected difference between authoritative/authoritarian and indulgent parents, it was used along with the nurturance subscale to assign mothers to the four general parenting styles. Therefore, mothers who chose the “setting rules” response above were classified as authoritative (42.8%) if they scored at or above the median on nurturance and classified as authoritarian (34.2%) if they scored below the median on nurturance. Mothers who chose the “learn from mistakes” response were classified as indulgent (11.8%) if they scored at or above the median on nurturance or classified as uninvolved (11.2%) if they scored below the median on nurturance. Olvera and Power [65], using a very similar sample, used median splits on the nurturance and the amount of control subscales to assign mothers to styles in their samples (alphas for the amount of control subscale in this study was 0.68). Given the relatively small percentage of mothers who chose the “learn from mistakes” response in this study (23%), the percentages of indulgent and uninvolved mothers in the current sample were considerably lower than in a the Olvera and Power study [65]: authoritative (19%), authoritarian (16%), indulgent (28%), and uninvolved (37%).

### 3.2. Validation of the PDI and General Parenting Styles

Because we used a new approach to assigning mothers to general parenting styles in the current study, it was important to demonstrate the validity of this approach. We did this by exploring differences on the non-feeding dependent variables as a function of general parenting style. We also conducted additional analyses to help validate the PDI in this population by predicting the non-feeding dependent variables from the four PDI parenting dimensions. Six of the ten regressions involving the four PDI dimensions were significant (*p* < 0.05). Multicollinearity was not a problem in that the Variance Inflation Factor (VIF) statistics were all less than 1.50. Examination of the standardized beta weights in the significant regressions showed that nurturance positively predicted three measures: positive social regulation (*beta* = 0.26, *p* < 0.01), effortful control (*beta* = 0.27, *p* < 0.01), and executive functioning (*beta* = 0.20, *p* < 0.05). Inconsistency positively predicted frustration (*beta* = 0.31, *p* < 0.001) and negatively predicted positive social regulation (*beta* = −0.17, *p* < 0.05) and delay of gratification (*beta* = −0.15, *p* < 0.05). Finally, organization negatively predicted frustration (*beta* = −0.17, *p* = 0.05), delay of gratification (*beta* = −0.24, *p* < 0.01) and emotional overeating (*beta* = −0.19, *p* < 0.05). Follow through on discipline was not a significant predictor in these regressions and no significant effects were found for food responsiveness, satiety responsiveness, eating in the absence of hunger, and child BMI *z*-score.

Three of the ten one-way ANOVAs showed significant effects of general parenting style: effortful control, *F*(3, 183) = 7.46, *p* < *0*.001, *eta*^2^ = 0.11; positive social regulation, *F*(3, 183) = 2.69, *p* < *0*.05, *eta*^2^ = 0.04; and eating in the absence of hunger, *F*(3, 182) = 2.62, *p* = *0*.05, *eta*^2^ = 0.04. Follow-up Bonferroni tests showed different patterns for the three analyses. Children of authoritative mothers (*M* = 6.07; *SD* = 0.54) showed significantly higher levels of effortful control than children of authoritarian (*M* = 5.59, *SD* = 0.75) or uninvolved (*M* = 5.66, *SD* = 0.56) mothers (*p* < 0.001 and *p* = 0.05 respectively). Children of indulgent mothers showed an intermediate amount of effortful control (*M* = 5.82, *SD* = 0.62). Children of authoritative (*M* = 3.75, *SD* = 0.34) showed significantly higher levels (*p* = *0*.05) of positive social regulation than children of authoritarian mothers (*M* = 3.57, *SD* = 0.50). Children of indulgent (*M* = 3.76, *SD* = 0.26) and uninvolved (*M* = 3.66, *SD* = 0.40) mothers did not differ from the other groups. Finally, although no Bonferroni tests were significant for eating in the absence of hunger, children of indulgent mothers showed the highest score for eating in the absence of hunger (*M* = 2.55, *SD* = 0.60). The means for the other groups were: authoritative (*M* = 2.11, *SD* = 0.75); authoritarian (*M* = 2.27, *SD* = 0.72); and uninvolved (*M* = 2.00, *SD* = 0.89). The Bonferroni comparison between children of indulgent and uninvolved mothers was marginally significant (*p* = 0.10).

Although the ANOVA for children’s BMI z-scores was not signficant, the means were in the same direction as the Olvera and Power [65] study, with children of indulgent mothers showing the highest z-scores (M = 1.35, SD = 1.17) and children of authoritarian mothers showing the lowest (M = 0.80, SD = 1.05). As in the Olvera and Power [65] study, children of authoritative (M = 0.91, SD = 1.27) and uninvolved (M = 1.07, SD = 0.76) mothers showed intermediate values.

### 3.3. Relationships between General Parenting and Maternal Feeding Practices

Presented in Table 5 are the Pearson correlations between the subscales of the Parenting Dimensions Inventory and the Comprehensive Feeding Practices Questionnaire. The correlations of the subscales within these two questionnaires were mostly small or nonsignificant. The two exceptions were the moderate positive correlations between nurturance on the PDI and the follow-through and organization subscales. General parenting was associated with maternal feeding, with almost two-thirds of the correlations between general parenting on the PDI and maternal feeding on the CFPQ being statistically significant. These relationships are further clarified by the regressions that examined the independent contributions of the four PDI subscales in predicting feeding practices. As in the previous regressions, multicollinearity was not a problem since the VIF statistics were all less than 1.50. All four of the regressions were significant (see Table 6). Examination of the table shows that nurturance was positively associated with healthy eating guidance and negatively associated with restriction for weight. Inconsistency was positively associated with restriction for weight and promoting overconsumption, and negatively associated with monitoring. Finally, following through on discipline was positively associated with restriction for weight and organization was positively associated with monitoring.

Two of the four ANOVAs examining differences in maternal feeding practices as a function of general parenting style were significant: monitoring, *F*(3, 183) = 4.21, *p* < 0.01, *eta*^2^ = 0.06, and healthy eating guidance, *F*(3, 183) = 6.50, *p* < 0.001, *eta*^2^ = 0.10. Follow-up Bonferroni tests showed that authoritarian mothers (*M* = 3.93, *SD* = 0.79) were significantly less likely (*p* < 0.01) than authoritative mothers (*M* = 4.37, *SD* = 0.77) to monitor their child’s food consumption (indulgent, *M* = 4.25, *SD* = 0.63; uninvolved, *M* = 4.30, *SD* = 0.60). In contrast, mothers with an authoritative parenting style (*M* = 4.41, *SD* = 0.56) were significantly more likely (*p* < 0.001) to provide their child with healthy eating guidance than mothers with an authoritarian (*M* = 3.98, *SD* = 0.62) style (indulgent, *M* = 4.22, *SD* = 0.70; uninvolved, *M* = 4.06, *SD* = 0.58).

### 3.4. Relationship between General Parenting and Maternal Feeding Styles

Table 5 shows that all four PDI subscales were significantly correlated with maternal responsiveness and that inconsistency was positively correlated with demandingness. The regressions for both demandingness and responsiveness were significant (see Table 7). As in the previous regressions, multicollinearity was not a problem since the VIF statistics were all less than 1.50. As shown in the table, inconsistency was positively associated with demandingness and negatively associated with responsiveness. Additionally, follow through on discipline was associated negatively with demandingness. The ANOVAs showed significant differences between general parenting styles and mothers’ reports of both demandingness and responsiveness during feeding: demandingness, *F*(3, 183) = 3.02, *p* < 0.05, *eta*^2^ = 0.05; responsiveness, *F*(3, 183) = 3.01, *p* < 0.05, *eta*^2^ = 0.05. Follow-up Bonferroni tests showed that mothers with an uninvolved general parenting style showed significantly (*p* < 0.05) lower levels of demandingness (*M* = 2.71, *SD* = 0.52) than mothers showing the authoritative (*M* = 3.13, *SD* = 0.64) style (authoritarian, *M* = 3.09, *SD* = 0.54; indulgent, *M* = 3.12, *SD* = 0.50). In contrast, a marginal Bonferroni test (*p* < 0.07) showed that authoritarian mothers (*M* = 1.18, *SD* = 0.14) showed less responsiveness than authoritative (*M* = 1.25, *SD* = 0.19) mothers (indulgent, *M* = 1.28, *SD* = 0.17; uninvolved, *M* = 1.24, *SD* = 0.13).

A cross-classification of general parenting styles with feeding styles is presented in Table 8. The chi-square statistic for this table was not significant, *X*^2^(9) = 11.71, *p* = 0.23. The only parenting style where there was a match between the general parenting style and the most common feeding style was authoritarian parenting. However, given that the authoritarian feeding style was the most common feeding style in the sample as a whole (see last row in the table), this match appears to be an artifact of the overall rate of authoritarian feeding in this sample of mothers.

## 4. Discussion

Despite problems with the amount of control scale on the PDI, the revised approach to measuring general parenting style developed for this study appeared to be valid. Analyses of this approach using measures of children’s self-regulation collected as part of the larger Hughes et al. [60] study showed findings consistent with the literature on general parenting style and children’s adjustment [79,80]—children of authoritative mothers showed the highest levels of self-regulation and social adjustment (i.e., positive social regulation and effortful control) and children of indulgent mothers ate the most kilocalories in the absence of hunger—a finding consistent with Olvera and Power’s [65] finding that indulgent general parenting among Hispanic mothers with low incomes is associated with higher levels of childhood obesity. Although the general parenting style ANOVA examining child BMI *z*-scores was not significant in the current study, the means for children’s BMI *z*-scores were in the same direction as in Olvera and Power [65] with children of indulgent mothers showing the highest BMI *z*-scores.

Regarding the main study questions, the findings for the relationships between the general parenting dimensions and feeding dimensions/practices were largely consistent with the hypotheses derived from the past research in this area. As predicted, nurturance was positively associated with both healthy eating guidance (correlations and regressions) and feeding responsiveness (correlations only); inconsistency was positively associated with both restriction for weight and promotion of overconsumption (correlations and regressions); follow through on discipline was positively associated with monitoring, healthy eating guidance, and feeding responsiveness (correlations only); and family organization was positively associated with both monitoring (correlations and regressions) and healthy eating guidance (correlations only). Three unanticipated findings (consistent across both the correlations and regressions) were: a positive relationship between follow through on discipline and restriction for weight; a negative relationship between inconsistency and monitoring; and a negative relationship between inconsistency and responsiveness. The first finding may reflect a tendency for mothers who are both consistent in their discipline and concerned about their child’s weight to consistently enforce restrictions on their child’s eating. This may be particularly true for Hispanic mothers who show more restriction of children’s eating than non-Hispanic mothers [56]. The latter two findings suggest that mothers who have difficulty consistently responding to their child’s misbehavior (due to, for example, fatigue, conflicting demands, or emotional distress) might have difficulty monitoring their children’s food consumption or using child-centered feeding strategies during mealtimes. Several previous studies show that maternal depression and anxiety are associated with the use of parent-centered feeding strategies such as pressure to eat and restriction [81,82,83].

There was less support for the hypotheses regarding general parenting styles. Only the hypotheses for authoritative parenting were consistently confirmed. As predicted, authoritative mothers showed the highest levels of healthy eating guidance and showed high levels of monitoring and feeding responsiveness. Also as predicted, indulgent mothers showed high levels of feeding responsiveness, as well. Finally, the hypotheses for authoritarian mothers involving restriction for weight, promotion of overconsumption, and feeding demandingness were not confirmed—instead, authoritarian mothers showed a lower level of monitoring than authoritative mothers. As discussed in the introduction, the lack of findings for restriction may be due to differences in the function or impact of highly controlling parenting in Hispanic compared to non-Hispanic families [54,55].

It is not clear why so many of the hypotheses for general parenting styles were not confirmed. One possibility is that the alternative approach to defining general parenting styles used here (necessitated by the poor reliability of the amount of control subscale in this sample) makes comparisons with previous studies difficult. The use of one item with limited variance to assess the amount of control in the current study appears to have resulted in low numbers of indulgent and uninvolved parents, which undoubtedly reduced power to find significant effects. Given the problems with the amount of control scale in this sample, clearly further work on this scale is needed.

Despite strong support of all of the hypotheses regarding the relationship between the general parenting dimensions and maternal feeding styles/dimensions, there was no one-to-one correspondence between the general parenting and feeding styles in this study. This replicates the results of the only other study addressing this question [23] that also found no such relationship. This supports the concept of domain-specific parenting first described for the feeding domain by Costanzo and Woody [84]. That is, parents may show different parenting styles in different domains due to domain-specific concerns, strategies, and external pressures [85]. However, the results of this study, along with the findings of Hennessy et al. [23], suggest that there is indeed some relationship between general parenting and feeding—just not enough to result in matching styles across the two domains.

The results of this study should be considered in light of its limitations. As a correlational study, it is not clear whether the relationships identified here reflect the influence of general parenting on feeding practices or vice versa. Although it is likely that the direction of effects was from general parenting to feeding practices, the reverse direction of effects is possible as well. Secondly, because the main research questions relied on examining the relationships between various maternal self-reports, rater effects may have accounted for some of the results. Unfortunately, all previous studies on this question have relied on self-report methods—the inclusion of multiple methods (e.g., observations, experimental tasks, focus groups, interviews) would have increased the contributions of the current study. Third, we only studied mothers—fathers undoubtedly have an influence on children’s eating behavior as well (e.g., [86]), so future research should include fathers as participants. Finally, this research employed a rather homogeneous sample—primarily first generation, urban Hispanic mothers who had been born in Mexico or Central America. Given the diversity of parenting behaviors among different Hispanic groups [51], it is important to replicate these results in other Hispanic populations as well.

## 5. Conclusions

This study demonstrates that the relationships between general parenting and feeding practices in Hispanic mothers with low incomes are very similar to those identified in other populations. The results were much stronger for general parenting dimensions than for parenting styles, possibly due to the measurement of parenting styles in the current study. Despite the numerous relationships identified between general parenting and feeding practices/dimensions, there was no one-to-one correspondence between parenting and feeding styles supporting Costanzo and Woody’s [84] concept of domain-specific parenting. Because it is undoubtedly harder to change general parenting styles than feeding practices, the current results have implications for addressing the problem of childhood obesity through parenting interventions. The findings suggest that one can go about changing specific feeding practices that promote healthy eating in children without having to change the parent’s general parenting style. Such a conclusion supports the value of continuing to develop and evaluate programs to address childhood obesity by promoting responsive feeding styles in parents of young children (e.g., [87]).

## Figures and Tables

**Table 1:** Consistent relationships identified between Baumrind and Black’s [11] three general parenting styles and parental feeding practices ^a^.

Authoritative General Parenting	Authoritarian General Parenting	Permissive General Parenting
(Numerous Maturity Demands Enforced through Responsive, Child-Centered Strategies)	(Numerous Maturity Demands Enforced through Authority-Based, Parent-Centered Strategies)	(Few Maturity Demands; Use of Responsive, Child-Centered Strategies)
Greater Modeling [13,14,15]	Less Modeling [13,15]	Less Modeling [13,14,15]
Less Pressure to Eat [14,16,17]	Greater Pressure to Eat [13,16,17,18]	
Less Use of Food as a Reward or Punishment [14,16]		
	Greater Restriction [13,17,18]	Greater Restriction [18,19]
		More Emotional Feeding [14,16]
		Less Monitoring [13,14,19]

^a^ Table only includes relationships found in two or more studies.

**Table 2:** Consistent relationships identified between the four feeding styles on the Caregiver’s Feeding Styles Questionnaire (CFSQ) [24] and general parenting dimensions on the Parenting Dimensions Inventory (PDI) [25,26] ^a^.

Authoritative Feeding	Authoritarian Feeding	Indulgent Feeding	Uninvolved Feeding
(Encourages Eating through Responsive, Child-Centered Strategies)	(Encourages Eating through Nonresponsive, Parent-Centered Strategies)	(Allows Child Eat What He/She Wants; Uses Responsive, Child-Centered Strategies)	(Allows Child Eat What He/She Wants; Uses Nonresponsive, Parent-Centered Strategies)
High Nurturance	Low Nurturance	High Nurturance	Low Nurturance
High Follow-Through on Discipline	Low Follow-Through on Discipline	High Follow-Through on Discipline	Low Follow-Through on Discipline
Low Inconsistency	High Inconsistency	Low Inconsistency	Low Inconsistency

^a^ Table only includes relationships found in both the Hennessey et al. [23] and Hughes et al. [24] studies.

**Table 3:** Characteristics of the sample.

Characteristic	*M* (*SD*) or %
	*n =* 187
Parent gender-female	100.0
Child gender-female	47.6
Child age in months	57.4 (5.2)
Education of parent	
High school diploma or less	64.7
Some college or more	35.3
Currently employed	23.5
Marital status	
Married	59.3
Never Married	14.4
Widowed, separated, or divorced	26.3
Immigrant status	
Born in the U.S.	21.4
Born in Mexico	60.4
Born in Central America	16.6
Born in another country	1.6
Child Body Mass Index (BMI) categories ^a^	
Healthy (<85th percentile)	52.9
Overweight (85th to <95th percentile)	20.9
Obese (≥95th percentile)	26.2

^a^ Based on BMI percentiles for children of the same sex and age.

**Table 4:** Descriptive statistics on all study variables.

Variable ^a^	*n*	Possible Values	*Mean*	*SD*
Parenting Dimensions Inventory				
Nurturance	187	1–6	5.36	0.57
Inconsistency	187	1–6	2.89	1.05
Follow-Through on Discipline	187	1–6	4.45	0.99
Organization	187	1–6	5.01	0.80
Comprehensive Feeding Practices Questionnaire				
Monitoring	187	1–5	4.20	0.76
Restriction for Weight	187	1–5	2.76	1.09
Promotion of Overconsumption	187	1–5	2.45	0.68
Healthy Eating Guidance	187	1–5	4.20	0.63
Healthy Eating Variety	187	1–5	4.68	0.55
Caregiver Feeding Styles Questionnaire				
Demandingness	187	1–5	3.07	0.59
Responsiveness	187	0.2–2.0	1.23	0.17
Child Body Mass Index (BMI) *z*-score	187	−4.0–4.0	0.94	1.14
Children’s Behavior Questionnaire				
Effortful Control	187	1–7	5.83	0.66
Emotion Regulation Checklist				
Positive Social Regulation	187	1–4	3.68	0.41
Frustration	187	1–4	1.72	0.49
Executive Function	184	−8.0–8.0	0.03	1.64
Delay of Gratification	180	1–3	1.90	0.92
Children’s Eating Behavior Questionnaire				
Emotional Overeating	187	1–5	1.68	0.62
Food Responsiveness	187	1–5	2.22	0.85
Satiety Responsiveness	187	1–5	2.84	0.65
Eating in the Absence of Hunger	186	1–3	2.20	0.75

^a^ High scores on all variables reflect high scores on the construct.

**Table 5:** Pearson correlations between subscales from the Parenting Dimensions Inventory, the Comprehensive Feeding Styles Questionnaire, and the Caregivers’ Feeding Style Questionnaire (*n* = 187).

Variable	1	2	3	4	5	6	7	8	9	10
1. Nurturance ^a^										
2. Inconsistency ^a^	0.01									
3. Follow Through ^a^	0.49 ***	−0.02								
4. Organization ^a^	0.41 ***	0.01	0.27 ***							
5. Monitoring ^b^	0.25 ***	−0.19 **	0.19 **	0.24 ***						
6. Restriction for Weight ^b^	−0.08	0.32 ***	0.15 *	0.03	−0.13					
7. Promotion of Overconsumption ^b^	−0.07	0.29 ***	−0.11	0.03	−0.14	0.14				
8. Healthy Eating Guidance ^b^	0.30 ***	−0.10	0.22 **	0.24 ***	0.21 **	0.12	0.08			
9. Demandingness ^c^	0.06	0.21 **	−0.10	0.14	−0.12	0.13	0.44 ***	0.12		
10. Responsiveness ^c^	0.24 ***	−0.25 ***	0.23 ***	0.16 *	0.28 ***	−0.05	−0.36 ***	0.24 ***	−0.50 ***	

^a^ Parenting Dimensions Inventory; ^b^ Comprehensive Feeding Practices Questionnaire; ^c^ Caregivers’ Feeding Style Questionnaire; * *p* < 0.05. ** *p* < 0.01. *** *p* < 0.001.

**Table 6:** Multiple regression predicting subscales of the Comprehensive Feeding Practices Questionnaire (CFPQ) from the four Parenting Dimensions Inventory (PDI) subscales (*n* = 187).

	Monitoring			Restriction for Weight			Promotion of Overconsumption			Healthy Eating Guidance		
	*R*^2^ = 0.12 *F*(4, 182) = 6.53, *p* < 0.001			*R*^2^ = 0.16 *F*(4, 182) = 8.93, *p* < 0.001			*R*^2^ = 0.10 *F*(4, 182) = 5.16, *p* < 0.001			*R*^2^ = 0.12 *F*(4, 182) = 6.41, *p* < 0.001		
PDI Subscale	*B*	*SE*	*Beta*	*B*	*SE*	*Beta*	*B*	*SE*	*Beta*	*B*	*SE*	*Beta*
Nurturance	0.20	0.11	0.15	−0.45	0.16	−0.24 **	−0.06	0.10	−0.05	0.22	0.09	0.20 *
Inconsistency	−0.14	0.05	−0.19 **	0.34	0.07	0.33 ***	0.19	0.04	0.29 ***	−0.06	0.04	−0.10
Follow through	0.06	0.06	0.07	0.28	0.09	0.25 ***	−0.07	0.06	−0.10	0.05	0.05	0.08
Organization	0.15	0.07	0.16 *	0.08	0.10	0.06	0.06	0.07	0.07	0.10	0.06	0.13

* *p* < 0.05; ** *p* < 0.01; *** *p* < 0.001.

**Table 7:** Multiple regression predicting measures of demandingness and responsiveness derived from the Caregivers’ Feeding Style Questionnaire (CFSQ) from the four Parenting Dimensions Inventory (PDI) subscales (*n* = 187).

	Demandingness			Responsiveness		
	*R*^2^ = 0.09 *F*(4, 182) = 4.34, *p* < 0.01			*R*^2^ = 0.14 *F*(4, 182) = 7.42, *p* < 0.001		
PDI Subscale	*B*	*SE*	*Beta*	*B*	*SE*	*Beta*
Nurturance	0.09	0.09	0.08	0.04	0.02	0.15
Inconsistency	0.12	0.04	0.21 **	−0.04	0.01	−0.25 ***
Follow Through	−0.10	0.05	−0.17*	0.02	0.01	0.14
Organization	0.11	0.06	0.15	0.01	0.02	0.06

*p < 0.05; **p < 0.01; ***p < 0.001.

**Table 8:** Cross-classification of general parenting styles from the Parenting Dimensions Inventory (PDI) and feeding styles from the Caregivers’ Feeding Style Questionnaire (CFSQ).

	CFSQ Feeding Style			
PDI General Parenting Style	Authoritative	Authoritarian	Indulgent	Uninvolved
Authoritative	15.0%	33.8%	40.0%	11.2%
Authoritarian	14.1%	40.6%	23.4%	21.9%
Indulgent	27.3%	36.4%	31.8%	4.5%
Uninvolved	14.3%	23.8%	38.1%	23.8%
Total	16.0%	35.3%	33.2%	15.5%

*X*^2^(9) = 11.71, *p* = 0.23.

## Data Availability

The data presented in this study are available on request from the corresponding author. The data are not publicly available due to privacy and confidentiality.
